# Bringing soil chemistry to environmental health science to tackle soil contaminants

**DOI:** 10.3389/fenvs.2022.981607

**Published:** 2022-09-15

**Authors:** Owen W. Duckworth, Matthew L. Polizzotto, Aaron Thompson

**Affiliations:** 1 Department of Crop and Soil Sciences, North Carolina State University, Raleigh, NC, United States; 2 Department of Earth Sciences, University of Oregon, Eugene, OR, United States; 3 Department of Crop and Soil Sciences, University of Georgia, Athens, GA, United States

**Keywords:** contamination, bioavailability, transdisciplinary, cross-disciplinary, collaboration, exposure

## Abstract

With an estimated five million sites worldwide, soil contamination is a global-scale threat to environmental and human health. Humans continuously interact with soil, both directly and indirectly, making soils potentially significant sources of exposure to contaminants. Soil chemists are thus a potentially dynamic part of a collaborative cohort attacking environmental health science problems, yet collaborations between soil chemists and environmental heath scientists remain infrequent. In this commentary, we discuss the unique properties of soils that influence contaminants, as well as ways that soil chemists can contribute to environmental health research. Additionally, we describe barriers to, and needs for, the integration of soil chemistry expertise in environmental health science research with a focus on the future.

## Introduction

**FIGURE 1:**
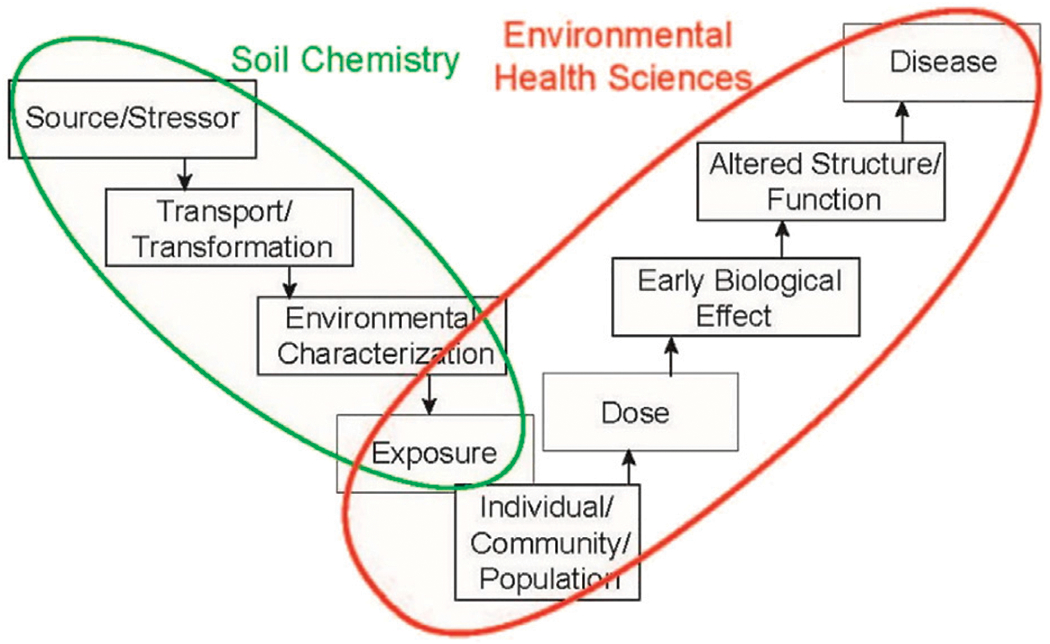
The environmental health research continuum. Green and red ovals indicate siloed domains that separate soil chemists from environmental health scientists. Redrawn from (USEPA, 2003).

Soil chemical processes play essential roles in human health by controlling the distributions of environmental contaminants and regulating how much and how rapidly these chemicals are transferred along exposure routes. These routes of exposure from soil are complex, and thus soil chemists can be integral parts of collaborative cohorts addressing environmental health science problems. The USEPA developed an exposure-dose-effect model to guide environmental human health research (Figure 1). However, this model has rarely moved from its use in government intuitions to academia, where a large proportion of soil chemistry research is conduced. We note that it is easy to silo the domain of soil chemists (green oval, left) from domain of environmental health scientists (red oval, right); consequently, many studies about environmental contaminants do not span the entire continuum and thus limit their actual impact. Despite an increasing emphasis on convergence approaches to solve societal challenges (National Science Foundation, 2016; National Academies of Sciences and Medicine, 2021), collaborations between research soil chemists and environmental heath scientists remain infrequent. Similarly, public outreach programs that typically have strong soil chemist input, such as the USDA agricultural extension service, have only recently begun emphasizing urban environments where agricultural activity is confronted with complex and heterogeneous soil contaminant distributions, which arise from the extreme social-ecological spatial heterogeneity (patchiness) of all urban and peri-urban systems (Byrne, 2021).

Soils are the living skin of the Earth (Brantley et al., 2017), and nearly all human activities are intimately entwined with soils. This not only means that humans are continuously exposed to soil, either directly or indirectly, and thus soil is always part of our exposure pathway, but also that soils may become contaminated from diverse human activities, such as accidental release of hazardous materials, the purposeful land-disposal of waste, and the deposition of particulates or solutes from the atmosphere. Globally, it is estimated that more than five million sites have soils contaminated with metals (Khan et al., 2021), with a recent report on global soil contamination (inclusive of metals, non-metallic inorganics, and halogenated and non-halogenated xenobiotic organic compounds) describing the situation as bleak (FAO and UNEP, 2021). Table 1 further highlights the scale of the problem with examples of contamination for broad geographic areas. Contaminated soils represent a significant source of chemical exposure to people through direct soil contact, inhalation of soil-dust particles, or soil ingestion, as well as indirect sources of contamination through air, water, or food (Brevik and Burgess, 2012).

Exposure to pollution has real consequences, causing an estimated nine million human deaths in 2015 alone (Fuller et al., 2022). Soil chemists and environmental health specialists must work together to better understand the forms and hazards posed by contaminated soil, define the scales of risks, create feasible mitigation strategies to minimize human exposure to soil contaminants, and improve human health. Here, we offer a commentary to highlight the need for engendering more collaborations, with a focus on engaging United States academia. We discuss the unique properties of soils that influence contaminants and discuss ways that soil chemists can better contribute to environmental health research. Additionally, we describe barriers to, and needs for, the integration of soil chemistry expertise in environmental health science research with a focus on the future.

## A soil chemist’s perspective: What are the unique properties of soils that impact contaminants?

Soil is “the most complex biomaterial on the planet” (Young and Crawford, 2004), comprising heterogeneous bio-physicochemical reaction pathways that are critical for human, ecological, and planetary health. Challenges in interpreting data and implementing remediation of contaminants in soils stem largely from the fact that true soils deviate from simpler aqueous or “clean sediment” systems from which most mechanistic information is derived.

At nanometer to centimeter scales, soils are comprised of inorganic and organic solid phases co-generated by plant roots, microorganisms, and chemical weathering reactions that can trap contaminants in pores and microsites, and expose them to reactive solid phases where they are immobilized or transformed (Yu, 2018). These soil components provide a diverse array of surfaces that may sorb or transform contaminants, governing their transport and lifetime in the environment. The inorganic solids include primary minerals (e.g., quartz, feldspars, biotite) and secondary minerals (e.g., 1:1 and 2:1 layer clays, Fe and Al oxides) spanning sand to clay-sized particles with correspondingly increasing specific surface areas and reactivities as size decreases (Sposito, 2008). This surface area/reactivity relationship with size places disproportionate emphasis on the short-range-ordered (SRO) iron and aluminum oxide phases, that are typically ≥10 nm in size and can dominate soil surface area and reactivity. These SRO phases are particularly important in high weathered or acidic soils, where other reactive solid phases like 2:1 clays are absent and SRO metal phases are often co-precipitated with organic matter (Kleber et al., 2015), generating nano-sized particles with a range of surface charges and sorption sites for both polar and non-polar contaminants (Thompson and Goyne, 2012). In alkaline soils, calcium and carbonate-bearing phases become more important both for accumulating organic compounds *via* calcium-bridging reactions, and *via* co-precipitation of metal contaminants, such as calcium-phosphate and lead-carbonate phases (Song et al., 2022). Soils are also replete with smaller, but critically-important organic-based solid phases in the form of soil organic matter (SOM). SOM may represent only a small percentage of the soil by mass or volume, but—particularly for non-polar contaminants—these phases can explain the bulk of organic contaminant behavior as they harbor essentially the only non-polar regions in the soil solid phase. When present, pyrogenic carbon (char or biochar) derived from natural or anthropogenic fires can be a potent, high surface-area solid phase that has abundant regions of non-polar sorption sites, and generally—but not always—has a long residence time in soils relative to other forms of SOM (Schmidt et al., 2011). In addition, ionizable functional groups on organic matter—most notably the carboxylic acid group—provide essential negative surface charge becoming the dominate cation adsorption sites in most soils without 2:1 clay minerals.

While these soil solid phases are important as sorbents and reaction centers for contaminants, it is their 3-D arrangement that structures much of the complexity in how contaminants interact in soils (Vogel et al., 2022). At the profile-scale, soils develop macro-pores along old root channels and animal burrows and in response to lithologic discontinuities that channel contaminants to deeper depths and expose them to variable conditions (Franklin et al., 2021). Within the soil matrix, a continuum of aggregate structures exists to generate progressively smaller pores, niches, voids, and microsites: from macro-aggregates that comprise blocky or platy soil structural elements to micro- and nano-aggregates (Totsche et al., 2018). These aggregate structures are typically cemented at various strengths either via organic biological exudates or SRO iron oxides. Contaminants migrating through the soil’s micropore structures can become trapped and inaccessible even without undergoing sorption reactions when pores become disconnected and orphaned from macropores (Franklin et al., 2021). Microorganisms, which can drive much of the organic contaminant decomposition or inorganic contaminant transformations, inhabit the fractal pore structure of soils unevenly, residing in regions with current (or past) concentrations of substrates that may or may not coincide with the contaminant distribution. This combination of multiscale complexity has profound influence on the behavior of contaminants that can monopolize the efforts of soil chemists, especially those seeking mechanistic insights into the fate of soil contaminants.

## How do soil chemists typically respond to contaminant risks?

Soil chemists are trained to embrace and focus on the transport and transformation of contaminants in complex, heterogeneous environments, and consequently have a detailed perspective and powerful toolkit for working on the left arm of Figure 1 traditionally, they are less well suited for expanding research into the right arm of Figure 1. However, soil chemists may directly reduce human exposure by improving soil remediation strategies. Strategies to remediate soils should both mitigate human exposure to contaminants and allow for beneficial re-use of the reclaimed site. Recent reviews have highlighted that, although commonly used remediation approaches can be effective, many involve removal (landfilling), hard stabilization (e.g., encapsulation, hard capping, solidification, and vitrification), or other approaches that substantially disturb soil function (e.g., soil washing and electrokinetic methods) (Sarkar et al., 2019; Khan et al., 2021). Less disruptive approaches such as biological extraction, stabilization, transformations (including bioremediation and transformation) may be effective only under certain conditions (Khan et al., 2021). Soil chemists can provide mechanistic insights to better understand and improve the efficiency of these approaches. Additionally, inorganic or organic soil additives (including various types of nanoparticles, sorbents, carbon sources, acids or bases, and oxidants or reactants) may modify biogeochemical processes to promote degradation or immobilization/deactivation of contaminants (Sarkar et al., 2019; Palansooriya et al., 2020). Continued development of these types of approaches—rooted in mechanistic insights—may allow for more widespread *in-situ* remediation with less landscape disruption and open remediation sites to more reuse options. To accomplish this, soil chemists will need to expand collaborations with environmental health scientists.

## What challenges limit soil chemist engagement with environmental health scientists?

An additional way in which soil chemists may reduce human exposure to contaminants is to extend their efforts and work across the environmental heath continuum (Figure 1). Although current soil chemist approaches that seek deep mechanistic understanding may be scientifically meritorious and may lead to improved radiative or predictive capabilities, these efforts do not often extend to “right arm” of Figure 1. How do soil scientists engage environmental health scientists, who focus on the sociological, public health, biomolecular, or medical aspects of human systems?

Despite the detailed knowledge gleaned from decades of soil chemical research, there remain limits to using soil chemical knowledge for quantifying risks of human exposure to soil contaminants. Standards or maximum allowable concentrations of elements, chemicals, or families of chemicals in water and air have been set for many toxicants by Federal and State governments. These are typically set based on models of exposure that may lead to increased risk of disease, but assume that some fraction of the toxicant in water or air is bioavailable, typically through ingestion or inhalation. However, the routes of exposure from—and bioavailability of contaminants in—soils are less straightforward, and generally estimates of bioavailability are based on animal models or *in vitro* extractions of a range of soils (Bradham et al., 2011). As expected, bioavailability of different contaminants varies with edaphic properties (Smolders et al., 2009; Bradham et al., 2011; NEPC, 2013), and it is challenging to conduct accurate exposure modeling and thus devise overarching health guidance related to soils (as compared to drinking water, which is often used as a reference for bioavailability). A case in point is soil lead, where health and remediatory guidelines in different countries worldwide span nearly four orders of magnitude (Jennings, 2013; Schwarz et al., 2016).

The compositional complexity and heterogeneity of soils makes it difficult to predict the concentrations, forms, and reaction pathways governing a soil contaminant’s risk at any one point in space and time. Although it is dogma among soil chemists that molecular-scale insights lead to improved management of soils, it is nearly impossible to identify the drivers of contaminant risks given the multitude of chemical, physical, and biological components that may each be acting on a contaminant and governing its phase, speciation, bioavailability, and mobility within a soil. Indeed, the currently used indices for contamination and risk are associated with bulk concentration (often normalized to a background) and do not account for other edaphic factors that control the speciation and mobility of contaminants (Kumar et al., 2019; Khan et al., 2021). Even when contaminant speciation is taken into account for risks or remediatory levels, such as is sometimes the case with toxic Cr (VI) versus total Cr in soils, analytical challenges limit effective quantification. Further, should contaminant risks for any given location be revealed with certainty, how they change over space and time must be quantified, as it is not uncommon for soil contaminants to vary over scales such that people in close proximity face substantially different contaminant exposure routes and risks.

Given these challenges, when pressed to give concrete information about soil contaminant risks to environmental health specialists, soil chemists end up relying on broad chemical indicators—typically pH and Eh—and simple macroscale chemical extractions to define the bioavailability and prospective exposure routes of soil contaminants. Moving forward, soil chemists need to more comprehensively evaluate the extent to which molecular-scale knowledge upscales to macroscopic measurements and predictions of contaminant risks to human and environmental health. Similarly, soil chemists need to develop better means for describing spatial and temporal heterogeneity in soil chemical properties as it relates to contaminant exposure risks, as well as quantify uncertainty in heterogeneity. As these approaches are developed, it is essential to better identify and target the needs of environmental health professionals that actively work to understand and limit human exposure to contaminants.

## Successes in linking soil chemistry and environmental health protection

Although the aforementioned barriers and challenges exist, successful collaborations between soil chemists and health scientist do exist and these can provide models for future collaborations. The USEPA, whose mission involves research that protects human health and the environment, has a long tradition of working across the framework in Figure 1. An example of these efforts is the USEPA Great Lakes Region’s Remediation to Restoration to Revitalization (R2R2R), which seeks to remediate contaminated areas to restore ecosystem services and revitalize communities (Williams and Hoffman, 2020). These projects engage diverse scientists, including soil chemists, ecologists, biologist, and health scientists, to work with stakeholders to convert contaminated lands to thriving areas. Beyond government labs, the NIEHS superfund research program (National Institute of Environmental Health Sciences, 2022) seeks to bring mostly university based researchers studying contaminants in the environment together with scientists and health professionals studying exposure and its disease consequences. These projects work on a wide range of topics and currently involve 120 universities. However, more and expanded programs are needed both domestically and internationally to tackle the daunting problem of soil contamination and its burden on human health.

In addition, intersections of health and soil science are quite obvious in the rapidly expanding urban agriculture sector, which places food production proximal to potential contaminant sources. Many urban farmers are first-generation agronomists with limited resources, operating diversified farms on soils that may have legacy contaminants (Pouyat et al., 2010). Although not yet widely available, several programs have been developed to facilitate soil testing, at the national [Australia (Taylor et al., 2021)], state, and local levels [Baltimore; (Schwarz et al., 2016)] Coupling these efforts with a greater infusion—and advertisement—of technical support for interpreting and restoring soils in urban communities for agricultural use is happening, but remains embryonic (Byrne, 2021).

## Future outlook and priorities: Bridging the soil chemistry-environmental health divide

Considering previous limitations and successes in linking soil chemistry and environmental health, we recommend four broad actions to better understand and respond to soil contaminants:

### Build systems to encourage information transfer among scientists

To better understand the needs of, and collaborate with, environmental health scientists, soil chemists must engage administrators and funding agencies to overcome structural impediments that limit the flow of information across the entire environmental health research continuum. Currently, many soil chemists work in agricultural colleges at land grant universities. At these institutions, interdisciplinary collaborations with health scientists are not generally visible (or encouraged) for many soil chemists and thus we need commitments by administrators to support, promote, and value broader, non-traditional interdisciplinary efforts by their faculty (Sá, 2008; Harris, 2010).

### Enhance funding for programs addressing soil contaminants from source to health outcome

A commitment amongst organizations that fund science will be needed. Currently in the United States, national large-scale funding that encourages soil chemists to collaborate with health professionals is limited to programs within the USEPA and the NIEHS (NIEHS, 2007; Landrigan et al., 2015). Convergence approaches adopted previously between NSF and USDA (e.g., soil carbon research or soil sensors) could be implemented between NIH and USDA to address grand challenges in environmental human health and encourage research bridges between environmental health and soil scientists. Additionally, funding partnerships between basic science funders (NSF), those interested in public health (NIH, USEPA, and USDA), and those that manage contaminated sites (USEPA, DOE, DOD, State Governments) could provide platforms for convergence human health research that incorporates fundamental soil science insights. Such programs should be focused on specific locations where contaminants are present and impacting environmental health, rather than mostly supporting basic research.

### Promote education in team science

A key emphasis moving forward must be on the education of students to be future leaders at institutions and universities, emerged in team science and trained to attack real work problems. Soil chemists must look for interdisciplinary models (Duckworth et al., 2017) that allow them to train students with multidisciplinary perspective while maintaining their unique disciplinary identity. This may include also include encouraging attendance at conferences from allied disciplines (e.g., American Chemical Society or Society of Toxicology and Environmental Chemistry) or conferences that bring scientists together with stakeholders and members of affected communities so that students may internalize the real-world impact of their work (e.g., National PFAS Conference). Participation in community engagement and service activities also may help to motivate students that have the potential to transform soil chemistry and better human health. Many of the current generation of soil scientists are uncomfortable or poorly equipped to translate science insights into policy or communicate them to the public (Brevik et al., 2020). In terms of policy, programs exist to help soil scientists learn more about advocacy (Polizzotto, 2016), but more widespread formal training is needed to maximize the impact of research for public benefit.

### Harness existing resources to communicate soil contaminant risks and responses to the public

Currently, there is no widespread systematic approach for communicating about soil contaminants to the public. The USDA Cooperative Research and Extension Services could be enhanced to serve this role as it falls clearly within their mission. The recently formed urban agriculture advisory committee signals policy shifts at USDA to incorporate more urban support as part of their traditional extension efforts (USDA, 2021); further expanding support beyond food production to general environmental health topics might require USEPA and USDA partnerships around this issue. Contaminated soils disproportionally impact the health of communities of color and low-income communities. Incorporating community and citizen-science methodologies that engage frontline community members in participation and decision making is essential for tackling environmental injustice and to match remediation efforts with local community land-use needs (Fernández-Viña et al., 2022).

As the scientific community and the broader world work to address societal grand challenges and sustainable development goals associated with human health, we believe it is critical for soil chemists to increase their participation in these team science efforts aimed at the betterment of the human condition. Only through interdisciplinary partnerships, enhanced by improved communication and education, can meaningful reduction in human exposure to soil borne toxic chemicals and human disease burdens be achieved.

## Figures and Tables

**TABLE 1:** Examples illustrating the extent of soil contamination in broad geographic areas.

Location	Extent of soil contamination
Africa	Widespread metal contamination from activities such as farming, gold mining, roads, automotive repair, waste disposal, and e-waste recycling (Fayiga et al., 2018)
Australia	As many 2,00,000 sites with contaminated soil (Langley, 2002); 13,00,000–17,00,000 tons of contaminated soil waste for remediation or disposal material (Plant, 2014)
Canada	3,595 priority contaminated sites identified (Treasury Board of Canada Secretariat, 2022)
Chile	590 potentially polluted sites identified (Ministerio del Medio Ambiente, 2018; FAO and UNEP, 2021)
China	~1/5 of arable land and 1/6 of total land contaminated (Zhao et al., 2015).
Europe	~3,40,000 contaminated sites (Pérez and Eugenio, 2018); 1.2 M km^2^ (28.3% of total land) is suspected of metal contamination (Tóth et al., 2016)
Mexico	981 sites registered between 2006 and 2013 (Secretaría de Medio Ambiente y Recursos Naturales, 2018; FAO and UNEP, 2021)
India	A meta-analysis of 92 papers found widespread contamination of cadmium, arsenic, and other metals (Kumar et al., 2019)
United States	Approximately 1% of land area (22 million acres) contaminated by potentially hazardous chemicals (USEPA, 2012); 73 million people, disproportionately from vulnerable and minority populations, live within 3 miles of a Superfund Site (USEPA, 2021)

## Data Availability

The original contributions presented in the study are included in the article, further inquiries can be directed to the corresponding author.
